# Paleomimetics: A Conceptual Framework for a Biomimetic Design Inspired by Fossils and Evolutionary Processes

**DOI:** 10.3390/biomimetics7030089

**Published:** 2022-07-05

**Authors:** Valentina Perricone, Tobias Grun, Pasquale Raia, Carla Langella

**Affiliations:** 1 Department of Engineering, University of Campania Luigi Vanvitelli, Via Roma 29, 81031 Aversa, Italy; 2 Department of Invertebrate Palaeontology, University of Florida, Florida Museum, Dickinson Hall, Gainesville, FL 32611, USA; tgrun@ufl.edu; 3 Department of Earth Sciences, Environment and Resources, University of Naples Federico II, Via Vicinale Cupa Cintia 26, 80126 Napoli, Italy; pasquale.raia@unina.it; 4 Department of Architecture and Industrial Design, University of Campania Luigi Vanvitelli, Via San Lorenzo, 81031 Aversa, Italy; carla.langella@unicampania.it

**Keywords:** bioinspiration, evolution, constraints, biomechanics, virtual palaeontology

## Abstract

In biomimetic design, functional systems, principles, and processes observed in nature are used for the development of innovative technical systems. The research on functional features is often carried out without giving importance to the generative mechanism behind them: evolution. To deeply understand and evaluate the meaning of functional morphologies, integrative structures, and processes, it is imperative to not only describe, analyse, and test their behaviour, but also to understand the evolutionary history, constraints, and interactions that led to these features. The discipline of palaeontology and its approach can considerably improve the efficiency of biomimetic transfer by analogy of function; additionally, this discipline, as well as biology, can contribute to the development of new shapes, textures, structures, and functional models for productive and generative processes useful in the improvement of designs. Based on the available literature, the present review aims to exhibit the potential contribution that palaeontology can offer to biomimetic processes, integrating specific methodologies and knowledge in a typical biomimetic design approach, as well as laying the foundation for a biomimetic design inspired by extinct species and evolutionary processes: Paleomimetics. A state of the art, definition, method, and tools are provided, and fossil entities are presented as potential role models for technical transfer solutions.

## 1. Introduction

The interdisciplinary approach that combines the observation and understanding of natural functional adaptations with their abstraction and translation into technological applications is known as “Biomimetics”, “Bionics”, or “Biomimicry” [1,2,3,4,5]. This approach reveals the possibility of taking inspiration directly from structures, principles, and processes of organisms, i.e., products of evolution, for the resolution of disparate and insidious life problems. Examples of this are numerous, including the Japanese Shinkansen bullet train inspired by the kingfisher beak, rudders and fans inspired by humpback whale flippers, and the auto-repairing varnishes produced with polyurethane and chitosan of crustacean carapace [1,6,7]. 

Extant species provide inspiration for most biomimetic applications. Although reasonable, this limits the enormous variety of life forms and “technical” solutions produced by natural selection in the past and no longer viable in the extant biota. For example, whereas current flying vertebrates use muscle power and feathers to produce wing upstroke and exploit ascending air currents to soar, pterosaurs used their leathery wings to fly with less energy expenditure, ameliorating flight energetics through natural selection [8]. In today’s elephants and giraffes, columnar limbs supporting exceptional body weight can be observed, but a closer look at the fossil records suggests that the aligning limb bones could have provided support for greater body sizes, as in the sauropod dinosaurs [9]. Although studying extant organisms allows observation of the phenotype kinematics and performance, recent technological advances in digital 3D reconstruction, computational imaging, and numerical simulation for palaeontological analysis have opened a new era: fossilised specimens with their morphological features and functioning principles, finely selected over millions of years of evolution, can now be analysed in detail [10,11]. The newly developed field of virtual palaeontology allows modelling shapes and functional performances of past organisms, revealing multiple facets of their phenotypic adaptations and diversities [12,13,14]. These details can be effectively transferred into a variety of applications. Hence, extinct species on par with extant ones can now be considered in the development of bioinspired solutions leading to the emergence of Paleomimetics, i.e., a biomimetic design inspired by extinct species and evolutionary processes. This concept was also introduced by Shyam et al. [15] with the name of “Paleomimesis”, together with the Periodic Table of Life (PeTaL), consisting in a tool based on artificial intelligence that supports the identification of biological solutions for human applications. Successively, Tihelka [16] reviewed the potential industrial applications of fossil-inspired technologies named “Palaeobiotechnology”.

Fossils are preserved remains, impressions, or traces of animals and plants embedded in rock [17]. They represent the only evidence of previous organisms allowing scientists to reconstruct the morphological changes over time as a direct effect of evolution. The knowledge acquired from fossil species is also essential to understand and define current flora and fauna. Palaeontology is not limited to merely observing and describing ancient forms, but also provides important data on the evolutionary meaning of forms and functions in present organisms [17,18]. Hence, palaeontology may offer important insights on form and function that can considerably improve the efficiency of biomimetic transfer by analogy of function, contributing to the genesis of new shapes, textures, structures, and functional models for productive and generative processes.

In this context, the present article introduces the conceptual framework for Paleomimetics, emphasising the potential contribution that palaeontology can offer to biomimetics, integrating specific methodologies and knowledge in a typical bio-inspired design approach. Moreover, definition, methods, and tools of the Paleomimetic approach are introduced, and different fossil entities are presented as inspirations for new technical transfer solutions.

## 2. Disciplinary Transfer of Knowledge

The synergic collaboration between different disciplines, e.g., biology, engineering, design, and material sciences, allows the development of innovative solutions that overcome the usual intra-disciplinary methodological limits [19].

In this study, the synergic collaboration between palaeontology and biomimetic design is explored. This relationship can be conceived as bidirectional: palaeontology can inform design with new notions, form–function relationships, and processes; *vice versa*, palaeontology can obtain advanced knowledge from technical analyses. Biomimetic research transforms knowledge into materials, constructions, systems, and processes allowing them to be accessible to society as well as leading to a more detailed understanding of past biological systems (reverse biomimetics) [20]. 

### 2.1. From Palaeontology to Biomimetics

Palaeontology identifies and interprets morphologies, known as phenotypes, and evolutionary pathways from ancient times. These interpretations can be used in Paleomimetics to identify and abstract functional structures and processes evolved and adapted to ancient environments (including biotic and abiotic factors). A series of conceptual considerations are discussed regarding: (1) evolutionary processes; (2) organismal design constraints; (3) evolutionary traps.

#### 2.1.1. Evolutionary Process: Case and Necessity

It has long been noted that animal groups (known as clades, i.e., species sharing a single common ancestor) are separated from one another by large morphological gaps, indicating that evolution did not produce, and probably cannot produce, all the ideal range of phenotypes engulfed in life form diversities; gaps thus cluster species into well distinct areas within the phenotypic space. The existence of these gaps is one central tenet of the *bauplan* concept, defined as the set of anatomical and functional traits shared by diverse taxa and developed in relation to their specific evolutionary history. A bauplan is more than a mere pattern of clustered occupation of the morphospace; it is the natural consequence of established developmental pathways and constraints that force organisms to acquire specific phenotypes. Although explaining organismal design has long cherished the parallel between natural selection and human-made implements, the resemblance between the two is superficial. In contrast to any technical project, the bauplan is not the result of an intentional activity, but rather it is the consequence of the combined effects of *chance and necessity* that are properties of evolution [21].

The Darwin–Wallace theory properly describes evolution as a change in heritable traits (phenotype) of extant organisms, which are subjected to random variations (genetic variations) and events (genetic drift) over generations. Subsequently, the environment with its abiotic and biotic factors determines trait selection. Hence, traits environmentally adapted and heritable are preserved, while the others are eliminated, resulting in an increase or decrease in the frequency of certain traits within a population [22]. Hence, “drawn out of the realm of pure chance, the accident enters into that of necessity, of the most implacable certainties” [22]. In addition to natural selection, there is another selective force known as “sexual selection” defined by Darwin as “the advantage which certain individuals have over others of the same sex and species, in exclusive relation to reproduction”. Consequently, some traits have been selected due to their ability to enhance directly or indirectly successful reproduction. The selection therefore depends on individual reproductive success (fitness) and is also “cumulative”, i.e., selected traits are further developed, and new traits can be generated and added to those previously selected, resulting in an increase in their complexity over time. 

Sometimes biomimetics is erroneously carried out starting from the assumption that organisms evolve towards optimised shapes, structures, systems, and processes in a specific environment. In this light, biological adaptive principles and logic are transferred by analogy of function into human artefacts, processes, systems, activities, and technologies [2]. Nevertheless, not all traits are functional and optimised in organisms; in fact, a series of constraints and imperfections characterise organismal evolution (see Section 2.1.1 and Section 2.1.2). The process of evolution is not based on perfection, but rather on a series of attempts. Periods of species diversifications (radiations), prosperous stability (such as during the Carboniferous period), and extinction crises alternated through geological eras. For instance, a series of curious experimental forms and solutions were produced during the Devonian period. These life forms that lived in a brief period were called “hopeful monsters” and defined as hypothetical, radically new phenotypes, or monstrosities, generated from major mutations that radically sensibly altered the developmental pattern pathways in organisms. These organisms could possess major innovations providing new adapted functional traits for a different environment compared to that of their immediate antecedents [23]. This concept was introduced in 1933 by geneticist R. Goldschmidt, providing a new theoretical framework of macroevolutionary leaps in addition to the gradual process expected by the Darwinian evolutionary theory, which consisted in many small adaptive changes [23], although subjected to diverse controversy [24].

Human design, as an intentional activity oriented to perfect forms, systems, and processes, seems to be opposed to the evolutionary process. Indeed, since the Industrial Revolution, human design has been dominated by manufacturing and mass production rigours, in which industrial standardisation is a guarantee of constant high performance, while imperfection a weakness. This has led to a progressive homologation in the economic, political, and cultural fields. This trend has been currently re-evaluated in design practice, favouring customisation and originality, and including imperfections and randomness. Today, imperfection is perceived as a precious factor especially when deriving from artistry and, therefore, from a process in which individual human factors intervene, e.g., personal interpretations and errors. Human factors, and thus natural factors, can lead to uncertainty, inconsistency, inaccuracy, and discontinuity. However, just as in nature, these traits can be considered as opportunities. In the Japanese tradition, an entire philosophy, known as “Wabi-Sabi”, is centred on the acceptance of imperfect, impermanent, and incomplete aspects of artefacts, as in those in nature [25]. These design principles include asymmetry, roughness, heterogeneity, anisotropy, and organic forms that also characterise natural systems and processes [26]. These principles, and an in-depth comprehension of evolutive processes, are currently leading to novel ways of thinking and projecting materials, artefacts, buildings, and construction methods. Cutting-edge examples are “structural materiality” and “material ecology” based on the study and design of products and processes environmentally integrated using bioinspired, form-generation algorithms and digital fabrications developed at the MIT Media Lab [19,27,28]. Additionally, randomness occurs more often than expected in design processes. Numerous innovations and discoveries came about without an intentional process or sequence, such as the 3D printed high-tech textiles generated by fabrication errors, but then selected to realise new types of high-performance flexible textiles [29]. The evolution of human design entails numerous analogies with organismal evolution [30], for which evolutionary processes can be considered models and mentors, including randomness and imperfections. The evolutive process can help to foreshadow the transformation of errors into new opportunities and adaptiveness. In the human world, “hopeful monsters” can sometimes be seen as unusual designs, cutting-edge innovations, and visions, such as the giant metal trapezoid *Tesla Cybertruck* or the *Mercedes-Benz* vision of the new bionic, self-driving, electric car. Another analogy can be found in natural sexual selection. Products are selected by the market and often this selection is not only based on good product functionality, but also on customer needs, habits, trends, and subjective criteria. A sort of “Darwinian sexual selection” occurs between users and products. In some cases, highly functional, but non-attractive traits, can negatively affect user choices. Consequently, the identification of customer preferences and relative attractive traits can lead to a powerful directional choice of the product. 

#### 2.1.2. Evolutionary Constraints

The biomimetic approach is based on a transfer of working principles or processes centred on an analogy of function from nature to technical systems. This approach is often carried out without considering the environmental context, origin, and historical evolution of the biological model, leading to a potential alteration in the functional significance of the traits, resulting in erroneous and/or distorted projects. A biomimetic example that does not emphasise the ecological and environmental contextualisation is the Mercedes-Benz bionic car: a concept proposed by Daimler Chrysler AG and inspired by the yellow boxfish (*Ostracion cubicus*, Linnaeus, 1758). The shape of the car was based on the hypothesised low drag coefficient and high stability of the boxfish shape. However, Van Wassenbergh et al. [31] argued that reduced drag performance in the boxfish is lower compared to generalised fish morphology. In addition, the box shape is more likely destabilising to enhance manoeuvrability. It was discussed that the boxfish uses the destabilising effect of its shape to promote manoeuvrability and to perform 180° turning manoeuvres with a near-zero radius in a forward locomotion. Biologically, this turning performance responds to spatially complex habitats where manoeuvring is a necessity.

**Figure 1:**
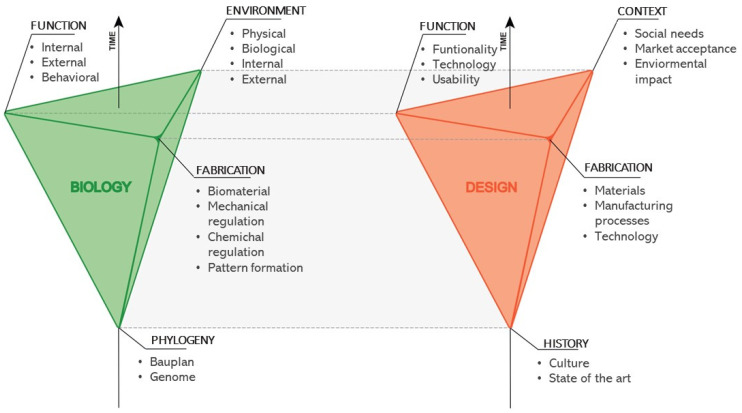
Morphodynamics in biology and design. Conceptual framework of evolutionary constraints in biology and design. Adapted from: Seilacher and Gishlick, 2014.

Besides the environmental context, it is important to consider that organismal design is not a result of an effective optimisation process or the best absolute solution in terms of functional efficiency; its evolution is determined by several constraints [18,32,33]. Accordingly, many organismal traits can easily invalidate the biomimetic transfer, constituting real traps for design projects (see Section 2.1.2). An in-depth understanding of the evolutionary, developmental (EvoDevo), and environmental contexts of the biological model is therefore necessary. In the constructional morphology framework, organismal traits are the result of several constraining factors influencing morphology and its evolution [18]. Observing biodiversity, it appears that every functional adaptation has already been generated; this is not always true, e.g., the wheel was not discovered in nature [18]. Indeed, diverse factors influence the development and maintenance of organismal traits. Constructional morphology identifies four key factors: phylogenetic, functional, morphogenetic fabrication, and environmental (Figure 1). The phylogenetic or evolutionary factor results from the organismal evolutionary history, transmitted as genetic heritage, determining the bauplan and representing the basic constraint for the development of new morphological traits or their variations. The biological functional factor is related to the proper internal, external, and behavioural (i.e., ethological) functioning of organismal traits and their adaptive significance, ensuring survival and fitness. The morphogenetic fabrication factor regards materials, biomineralisation, and growth processes. Organismal morphology is influenced and limited by biomaterial types and availability, physical-chemical formation processes, growth, and development. The effective environmental factor is determined by the biotic and abiotic environmental factors, which select organismal morphology, growth, and evolution. 

In palaeobiological practice, the constructional morphology framework provides a useful scheme in the formulation of basic questions by which organismal forms can be investigated. In this regard, it is intuitive how different species can develop similar morphologies and traits, even though they are phylogenetically distant. This phenomenon is known as “convergent evolution” (or “adaptive convergence”) and could be conceived as a common adaptive solution generated in response to similar and specific environmental factors and needs. In this case, selective pressure conducts to the development of common designs and adaptations. Examples are wings, which developed independently in various animal taxa, e.g., in pterosaurs (Sauropsida), bats (Mammalia), insects (Insecta), and birds (Aves), as well as the streamlined body shape, developed in Ichthyosaurus (Sauropsida), sharks (Chondrichthyes), pinguins (Aves), and cetaceans (Mammalia), which reduced fluid dynamic drag [34,35,36]. In plants, interesting convergent functional principles and mechanisms also developed; seed and fruit dispersal adaptations are outstanding examples. Indeed, during their evolution, many plant species developed anatomical structures to exploit winds (anemochory), water (hydrochory), and even animals (zoochory) for their expansion dispersal over great distances [37]. The earliest angiosperms did not exhibit many specialised modifications; during the Paleogene, fruits and seeds became more variegated, exhibiting specialised adaptations to enhance dispersal by means of winged fruits and seeds, fleshy fruits, and nuts [37]. Remarkable examples are feathery pappus that functions as a “parachute” (e.g., *Tragopogon pratensis* or *Sonchus oleraceus*) or winged achene, e.g., *Alsomitra macrocarpa* or *Acer circinatum* having helical motion able to swirl, similar to a propeller [38]. Convergent evolutive designs can thus be seen as valuable solutions for effective biomimetic transfer.

Biomimetic abstraction involves the translation of biological principles and processes into technical and design schemes from which a series of solutions and design-oriented concepts can be elaborated considering technical boundary conditions and limits. The concept of “constraints” or “boundary conditions” is of crucial importance in all solution transfers, being valid for both natural and technical systems. Accordingly, the evolutionary constraints diverge from the common vision of organisms as “elegant and perfect products” rather than projects based on compromises concerning available materials and resources, as well as difficulties in integrating different body plan components [35]. Nonetheless, paraphrasing Stephen J. Gould [39,40], these constraints are not only negative limits associated with structural restrictions preventing natural selection in an efficient trait modification but can also be seen as important factors in guiding organisms to adequate evolutive changes using only available sources [39,40]. The French biologist François Jacob stated that nature is an excellent tinkerer, not a divine artificer; thus, its design is not invention, but adaptation [41]. Similarly, what we learn from nature is not an absolute method in the development of something never created before, but the ability to adapt solutions to technology and designs already available, developing them into new functional ones.

In this perspective, a technical product can be considered a system that must “adapt” to several constraints, i.e., boundary conditions. Following the same scheme of constructional morphology, a product design can “evolve” from the interaction between several key factors: history, function, fabrication, and context (Figure 1). The historical factor depends on the design culture and the state of the art in its specific sector. It influences the development of the product and its “evolution” up to the present, determining its shape and function and representing the basic scheme for the development of new functional aspects or modifications. This factor can be evaluated through the analysis of technical necessity, product design history, state of art, and market analysis. The functional factor is related to the proper functioning of the product, including all internal and external mechanisms (e.g., mechanical, electrical, and chemical systems contributing to the functioning of the product). This factor also reflects the functional adaptation of the product to changing technology, societal needs, lifestyles, functionalities, and usability. Products acquire and lose details in their configurations according to expressed and unexpressed functional necessities. The fabrication factor concerns the manufacturing processes, i.e., the limits imposed by materials, fabrication practices, and technology. For example, the manufacturing process of aluminium restricts the shape of the final product compared to the introduction of 3D printing, which permits the realisation and proliferation of highly complex organic shapes (e.g., anisotropic porosity, honeycombed structures, hierarchical and layered morphologies), otherwise impossible to obtain using previous traditional methods [42,43]. The contextual factor is determined by societal and market influences that constrain product shape, working principles, fabrication, and “evolution” (e.g., interior or exterior design destination, industry and market requirements, society preferences and trends). As environment is the selection factor for organisms, so social needs and market (i.e., users) accomplish the final selection of newly developed designs determining their success and replication (e.g., from limited to mass production). Fortunately, the environment is also assuming a central role in product selection in terms of eco-compatibility and environmental impacts to assure a sustainable development. In this regard, the inspiration from natural systems and processes has a great potential for effective transitions from a human-centred to an environment-centred design [44]. The use of these boundary conditions can provide designers with a critical and operational framework on which they can carry out the analogical steps necessary for the transfer from natural to artificial dimensions.

Interestingly, a convergent evolution can also occur between biology and design in which organisms and human constructions have independently developed the same design strategies [45]. An example is the corrugated shell, a well-known strategy in structural engineering that is also found in seashells. Corrugation provides thinner, but stronger and stiffer shells, reducing material employment [46]. One of the earliest and famous applications of this concept on a large-scale structure is the Aircraft hangar at Orly Airport, Paris, by Eugene Freyssinet (1921) and the CNIT roof in Paris by Nicolas Esquillan (1958). The same strategy is present in bivalve shell adaptations, such as those in *Acanthocardia*, *Pecten*, and *Tridacna*, to optimise structural performance and material sources [47]. Another example is the geodesic dome, a hemispherical lattice shell that combines the structural efficiency of a triangular beam with a spherical shape, and is able to withstand heavy loads respect to their size. Patented by Buckminster Fuller in 1954, it was acclaimed as one of the main architectural discoveries of the 1950s, although it had already been adopted by Radiolaria about 540 Ma. These convergences derive from the fact that artefacts and organisms are often faced with similar problems and constraints, being both subjected to the same physical laws of the natural world [47,48,49,50]. 

#### 2.1.3. Evolutionary Traps

Due to the evolutionary process and constraints, organisms show constructions that are adapted to various environmental factors. These adaptations are adequate to survive, but given other constraints, organismal design can present traits that are imperfect, limited by other traits, or with no apparent functional role. Consequently, these traits can be easily misinterpreted, invalidating biomimetic transfer and constituting traps for design projects. Examples of these transfer traps are trade-offs, vestigial traits, sexual traits, and imperfections.

##### Trade-Offs

Since evolution regards the entire organism, each trait is not free to independently evolve. Variations in one trait could negatively affect other related ones. Subsequently, an organism is the result of a compromise between the functions of multiple traits. This compromise is defined as a “trade-off” and occurs when a beneficial change in one trait is associated with a detrimental change in one or more traits [35]. This is often due to the limited availability of resources (e.g., energy, space, time, elements). If two functions involve radically different requirements, with respect to the organismal design, the performance optimisation of one component may have a negative effect on the performance of another. For instance, trade-offs frequently appear in locomotory systems (e.g., muscle type, skeletal proportions): high speed and acceleration, useful for prey capture and predator evasion, can conflict with endurance or manoeuvrability (development of explosive speed force *versus* sustained endurance force); adaptation in climbing activities can be in conflict with level-running abilities [51]. Animal limbs optimised for speed with a lightweight and thin bone structure may reduce structural strength, resulting in easily fractured bones compared to shorter and massive ones [52]. Trade-offs can therefore represent important traps for a biomimetic transfer and the identified form–function could be an underdeveloped or sub-optimal functional trait in a compromise with one another. Hence, an in-depth knowledge of the biological model and its evolutionary history is necessary to understand and discern these traits. Trade-offs and their genetic bases can be presently identified and analysed through experimental manipulations of a trait and the observation of their effect on other trait modifications [35]. The in-depth understanding of trade-offs could also be seen as an opportunity, since the identification of structures with a fine balance between conflicting functionalities can be extremely useful to inspire multifunctional structures.

##### Vestigial Traits

Vestigial traits, also termed “evolutionary relicts”, are traits that no longer have a function in organisms and tend to atrophy; nevertheless, since they do not affect the organismal survival and fitness, these traits remain as an inherited trait [53,54]. Examples are pelvic bones in cetaceans and snakes, vestigial eyes in deep sea and fossorial animals, or the enormous, disproportionate egg of the kiwi bird [35,55]. Other examples can also be found in the human body: the plica semilunaris as a vestige of the nictitating membrane; the wisdom tooth reflecting a more prognathic face in the past [56]; and the tailbone, a relic of a past tail. Accordingly, a superficial observation of shells, bones, or other organismal traits is not sufficient to discern functional from non-functional traits and an in-depth historical analysis of the organism and relative experimental investigations can often lead to the identification of these evolutionary relics. Interestingly, a vestigial trait can also assume a new function through evolution. For example, facial expressions have been identified by Darwin as vestiges with respect to their original function and are currently selected for their intraspecific communication function [53]. Generally, a trait that shifts from a function to another during evolution is called “exaptation”, as proposed by Gould and Vrba [57]. In this regard, the bird feathers are a common example, taking into consideration that the initial development of feathers in their ancestors was for thermal insulation and not for flight [57].

##### Sexual Traits

Numerous phenotypic traits have evolved to provide advantages during courtship and mate choice. These traits are often expensive and decrease organismal survival, such as large bone crests, long feathers, huge antlers, bright colours, and extravagant behaviours. Two outstanding examples are the extravagant peacock tail or the enormous antlers of the Irish elk *Megaloceros giganteus*, a deer of the past. These sexual features demonstrate that the partner has sufficient resources to develop expensive traits. The cost of these traits includes not only the time and energy required for their construction and maintenance, but also a greater vulnerability to predators [58]. Sexual traits can be apparently considered paradoxical, in contrast to the well-known optimisation function of natural selection. In fact, they result from another important evolutionary selecting force: sexual selection (see definition in Section 2.1.1). Consequently, although sexual traits can shorten organismal life, they can directly or indirectly enhance individual reproduction (fitness).

Sexual traits can therefore deceive biomimetic projects: a sexual dimorphism, for example a bone thickening, could easily be mistaken for a functional characteristic. An in-depth knowledge on the bauplan and ethology of the biological model is necessary to understand and discern these traits.

##### Imperfections

Natural systems and processes are characterised by diffused examples of “imperfections”, sometimes referred to as an “unintelligent design” [59,60]. As stated by Pievani [61], this is because evolution works “not with the precision of an engineer who systematically optimises his inventions, but with the imagination and creativity of a craftsman, who transforms the available material as best he can”. Some evident examples of imperfection can be found in: (1) the laryngeal nerve of the giraffe, connecting the larynx to brain; instead of following a straight path, it passes from the brain to the chest, loops around one of the main arteries of the heart and then returns up to the larynx [60]; (2) the vas deferens, i.e., the duct of the vertebrate male transporting sperm from the testis to the penis, has a long detour around the ureter [60]; (3) the physiological blind spots in the human eye due to the passage of the optic nerve across the retina to reach the brain (an imperfection that does not occur in squids and octopi characterised by similar eyes) [62]. Imperfections are the direct result of compromise between organismal constraints (see Section 2.1.1) and can be explained by evolutionary history [60]. Once bauplan features and working principles are developed, it would be expensive and impossible to reset the genetic instruction and start from zero to produce a new, adapted bauplan. Consequently, imperfections emerge in the organismal design. These imperfections are tolerated as long as they do not lead to drastic negative effects or can be compensated. Additionally, imperfections can also result from other random events, such as drastic environmental changes. In this regard, an optimal trait can be transformed into an imperfect one through time: e.g., the huge dimension of sauropods had advantages in predation defence, thermal inertia, extended longevity, increased intelligence, and success in intra- and interspecific competition, but also disadvantages made them more susceptible to extinction with increased food and water requirements, developmental time, and lower fecundity [63,64]. 

### 2.2. From Biomimetics to Palaeontology

#### 2.2.1. Reverse Biomimetics

Biomimetic processes can lead to successful transfers and unique applications, but the development of biomimetic products also helps to improve the understanding of biological models, their bauplans, and evolutionary processes. In this context, the extracted functional principles and abstracted models of extinct species (which often can include 3D reconstruction and finite element analysis) are repeatedly evaluated, enhancing their knowledge [10].This process is referred to as ‘reverse biomimetics’ and can be conceived as an interactive spiral, in which the results achieved during biomimetic research can lead to a more detailed understanding of extant and extinct organisms, representing the basis for further investigations and eventual new transfers and developments of biomimetic products [20,65]. Biomimetic research can contribute to scientific progress itself, sustaining the advancement of palaeontological knowledge. Indeed, once the functional principles underlying extinct biological forms, structures, and compositions have been identified, the biomimetic design proposes to experiment and validate the acquired knowledge by recreating it in artefacts and processes, identifying the validity of the hypothesised, functionally inspired strategy. 

#### 2.2.2. Virtual Palaeontology and Digital Processes

Contemporary advances in computational imaging acquisition, numerical simulation, and manufacturing, together with an increased instrumental resolution for the analysis of extant and extinct species, have led to new developments for inter-disciplinary palaeontological studies and biomimetics. Fossil structures with their functional principles can be digitally analysed in-depth at a micro- and nanoscale [10,66] and thus can be better transferred into a multitude of constructions and industrial products. Consequently, fossils can be converted and analysed as 2D/3D models and directly connected to the technical process, becoming archetypes and/or guides for the development of artefacts and systems. This creates a supporting process with efficacious tools for designers, engineers, and palaeontologists in the recreation of functional shapes involving digital manufacturing techniques, which in a rigorous and functional way reproduce the analogous adaptations, even in extinct species [13].

Extinct organisms can be introduced in the technical and design world in the form of 2D/3D models to be carefully investigated, verified, and tested by means of advanced digital techniques. The functionality and performance of the different components can be highlighted through different types of digital numerical simulations, e.g., mechanical, fluid dynamic, optical, and thermal [10,14]. This process is carried out through several phases, ranging from the description and numerical analysis of details and elements of extinct biological models to the identification and translation of past biological knowledge in physical-mathematical inferential schemes, up to the concrete realisation (or synthesis) of such schemes in Paleomimetic artefacts [67].

**Figure 2:**
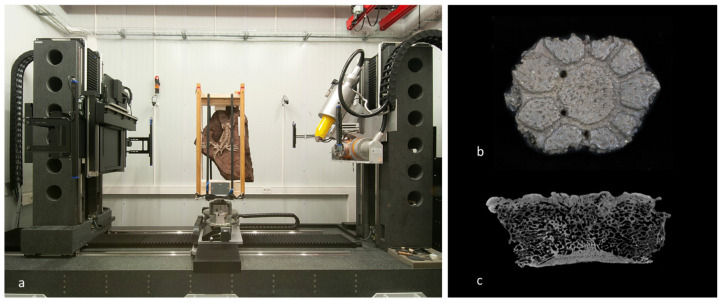
Virtual palaeontology: Computed Tomography. (**a**) Orobates fossil CT scan. Image courtesy of Tanja Kirsten, Technische Universität, Dresden and John A. Nyakatura, Humboldt University, Berlin. Osteoderm in (**b**) dorsal view and (**c**) Micro-CT scan transversal view. Image courtesy of Chris Broeckhoven, European Space Agency, ACT, Netherlands, and Anton du Plessis, Object Research Systems, Montreal, Canada.

Presently, it is possible to develop this Paleomimetic design process due to recent technological advances that provide (i) high fidelity in the capture of past models, (ii) greater reliability of results from numerical simulations, and (iii) high reproducibility of complex computer-aided design (CAD) models with digital manufacturing techniques [68,69]. High-fidelity 3D CAD models of fossilised structures can be acquired using various techniques, such as structure-from-motion (SfM) digital photogrammetry, 3D laser scanning and computed tomography, or microtomography (μCT) [11,70,71,72]. Each technique differs in geometric and volumetric resolutions of the model provided; thus, the choice of a technique greatly varies in relation to the investigation necessary. SfM photogrammetry, a computer vision technique, allows the recreation of a fossil surface in 3D CAD format using 2D digital photographs [70,72]. This reconstruction process can be completely automatised by specialised software (e.g., Agisoft PhotoScan or 3DF Zephyr) directly producing a 3D CAD millimetric geometric resolution. However, depending on the method used and personal expertise, better quality models can also be obtained when compared with the more automated techniques. For example, 3D laser scanners provide the direct acquisition of 3D geometric models of surface samples with resolutions ranging from a centimetre to a sub-millimetre [73]. By rotating the laser scanner, the position of hundreds of thousands of points are detected, defining the biological structure surface. Based on the detection speed and rotation steps, it is possible to control the scanning resolution [70], i.e., the density of the points detected at a defined distance, as well as the quality of the data acquired: the slower the rotation, the higher the resolution [74]. In the literature, comparisons between photogrammetry and laser scanning are available, e.g., [72,75]. Computed tomography provides more detailed reconstructions of samples through the acquisition of a series of sliced structures, organs, and tissues based on their density, which are detected using an X-ray beam [11] (Figure 2a). A high-resolution micro-computed tomography can scan samples of ten or less micrometres (Figure 2b,c). By combining the various sliced sections with ad hoc reconstruction software, it is possible to obtain a geometric (geometry-based reconstruction) and volumetric (voxel-based reconstruction) 3D model of the analysed sample [76]. 

Biomechanical investigations, which provide key knowledge regarding the structure–function relationships of extant and extinct biological systems using notions, methods, and tools derived from static and dynamic mechanics, are particularly promising for paleomimetic transfers [47,49,77]. In this framework, the term “function” refers to adaptive structural biological traits that increase the organismal fitness as well as the modalities of how these traits work and what “role” they absolve in the organism [77]. In mechanics, “statics” investigates forces acting on physical systems under equilibrium conditions and their consequent effects, whereas “dynamics” concerns the study of forces and their effects in motion. In this regard, FE analysis is an important technique for the reconstruction of stress, strain, and deformation in a digital structure that can be effectively used for biomechanical studies of extant and extinct organisms [10,78]. It allows palaeontological investigations in response to questions regarding organismal morphology, function, and evolution [10]. The main limitations of working with extinct organisms is that usually only fragmented component parts of the organisms are available e.g., [79], none or limited behavioural data are accessible, and their FE models cannot be directly validated [80]. Hence, palaeontologists commonly apply the principles of the “extant phylogenetic bracketing” (EPB) method [81], validating the models of closely related extant species to hypothesise the missing data and identify the main input parameters for their models. In this regard, Bright [80] provided a critical review on FE limitations in palaeontology, highlighting how the impossibility of accurately defining the original material properties of fossil material may impair the prediction of absolute performance values in palaeontological FE models, such as the skeletal breaking stress. Therefore, he reported that more material properties data should be collected from a range of tissues in a range of taxa to better understand the existing variation and select the most appropriate values for palaeontological FE models. Moreover, he reassuringly concluded that the FEM advantages lie in the ability to provide reliable patterns of stresses and strains even with limited input data (paying particular attention to the result magnitudes and trying not to over-interpret them), to effectively make relative comparisons between models, and to quantitatively assess how evolutionary changes in shape result in functional adaptations. Indeed, besides their limitations, computer-aided visualisation, image analysis, and FE models undoubtedly suggested interesting information about extinct taxa and past form–function relationships [82]. One of the first examples of FEA in palaeontology is a study carried out by Daniel et al. [83]. By analysing water depth pressures on ammonites, they questioned the previous assertion stating that increased ammonite septa complexity through time allowed the increased habitat depth occupation. This study was later disproved by a more detailed ammonite FE model, which confirmed the increased sutural complexity associated with depth [84].

Presently, new tools for dynamic systems are emerging. An example is multi-body dynamics (MBDA), a computational technique utilised in engineering applications and employed in biology and palaeontology to investigate the skulls of extant and extinct species [85,86,87,88]. This analysis allows the simulation of dynamic actions (e.g., feeding simulation) and the estimation of key biomechanical parameters that are often difficult or impossible to measure experimentally, such as: muscle activations and forces, joint reaction forces, bite forces, and jaw movement [85]. Successively, the predicted skull biomechanical loading can also be used in conjunction with FE analysis to further investigate the form–function relationship [85]. Bates and Falkingham [86] used dynamic musculoskeletal models to simulate maximal biting of the famous *Tyrannosaurus rex*. They estimated that an adult *T. rex* could sustained “bite forces of 35,000–57,000 N at a single posterior tooth, by far the highest bite forces estimated for any terrestrial animal”. Additionally, combining MBDA, machine learning algorithms and FE stress analysis, Sellers et al. [87] reconstructed the maximum locomotor speed in *T. rex*, demonstrating that its relatively long limb segments would have mechanically limited them to walking gaits.

An additional example of FEA and MDBA combination was provided by Lautenschlager et al. [88], who investigated three representative herbivorous dinosaur clades (*Plateosaurus engelhardti*, *Stegosaurus stenops*, *Erlikosaurus andrewsi*), demonstrating that despite the high degree of morphological skull similarity, their biomechanical behaviours were notably different, probably reflecting their dietary specialisations.

Other biomechanical analyses can be carried out to understand how ancient organisms moved in their past ecosystems. One method is computational fluid dynamics, simulating fluid flows of liquids or gases and their interactions with a solid body [89]. This is becoming increasingly important for the functional evaluation of flying and swimming animals. In the literature, few interesting examples are present (see [89] and reference cited therein). Using computational fluid dynamics, Dynowski et al. [90] investigated different feeding postures to analyse flow patterns forming around the *Encrinus liliiformis* crown, which lived in the middle Triassic.

**Figure 3:**
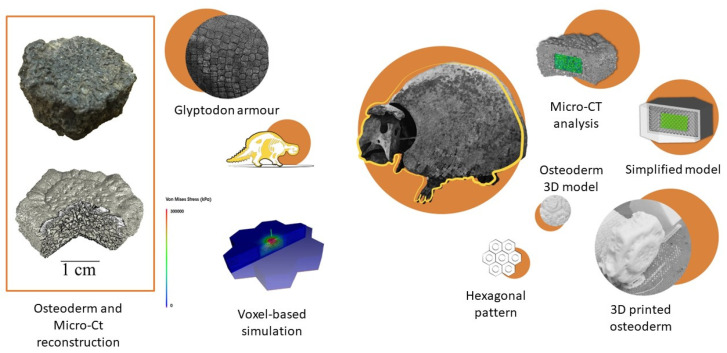
Virtual palaeontology: Micro-CT scan, reconstruction, analysis, and 3D printing. The *Glyptodon* osteoderm structure was reconstructed, analysed using FEA and recreated in simplified reverse engineered models to be mechanically tested. Graphical representation: Carmen Cerere. Images courtesy of Chris Broeckhoven, European Space Agency, ACT, Netherlands, and Anton du Plessis, Object Research Systems, Montreal, Canada.

In virtual palaeontology, an additional interesting tool is the use of additive manufacturing, including 3D printing, which could also be a pivotal instrument to characterise and replicate structures for future Paleomimetic transfers [68,69]. Three-dimensional printing is based on a computer-controlled process creating volumetric objects by depositing layers of material [91]. This process is generally flexible and allows the fabrication of a CAD model with good resolution and control on the resulting geometric shape [68]. Digital models of fossil entities obtained from the photogrammetric, light, and CT scan reconstructions can be 3D printed, creating physical models that can be scaled, observed, and/or tested, allowing an in-depth analysis of morphological and structural details. In this regard, an interesting example is the study of *Glyptodon* (early Miocene–Late Pleistocene), extinct mammals of the Cingulata order, which includes modern armadillos [69] (Figure 3). *Glyptodon* carapace consisted of osteoderms with a lattice core sandwiched between two compact layers. Starting from a micro-CT, the structure was reconstructed and recreated in simplified, reverse-engineered models. These models were 3D printed and tested by varying lattice and shell parameters. Results demonstrated that shell thickness was optimised compared to lattice density and lattice strut thickness [69]. Three-dimensional printing is useful not only for the study of extinct and existent organisms, but also for the opportunities to mimic their complex multiscale, multimaterial, and multifunctional structures [68]. Recent advances in material, process, and instrumental developments have enabled the evolution of additive manufacture processes to evolve from prototyping to manufacturing. Accordingly, numerous 3D-printed biomimetic materials, structures, and devices inspired by organismal design with mechanical, optical, electric, interactive, and adaptive properties have been developed. Fascinating examples are reported in the review elaborated by Yang et al. [68] and du Plessis et al. [69], in which they provide examples of recent progress and different forms of biomimetic engineering now possible using these technologies. 

#### 2.2.3. Valorisation of Basic Palaeontological Research

Transferring the results of palaeontological research to biomimetic projects, biomimetics provides achievements and benefits of scientific discoveries directly accessible to society. The basic palaeontological research, aimed to accomplish advancements in knowledge and understanding of extant and extinct organisms, is converted into applied research that translates results into biomimetic products. Due to the introduction of technologies, such as 3D CAD modelling, simulation, and additive manufacturing, traditional palaeontological research can be converted into innovative and advanced processes. This has important implications, mainly in research investment programs. Exploratory studies induced by the curiosity and intuition of scientists could provide new funding possibilities from companies and public and private institutions. 

## 3. Fossil-Inspired Design: The State of the Art for Launching Paleomimetics

**Figure 4:**
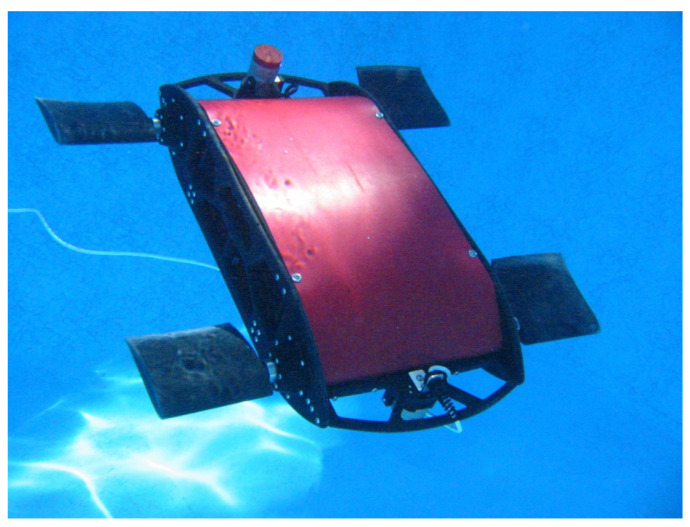
Madeleine, the aquatic robot imitating tetrapodal swimming. Image courtesy of John H. Long, Vassar College, USA.

In the literature, few examples of fossil-inspired designs are available, predominantly in the field of robotics [92,93,94,95,96] and flight technology [97,98,99,100,101,102]. One of the first examples is “Madeline”, a self-contained and self-propelled underwater vehicle with four independently controlled flippers, inspired by the evolutionary transformations of terrestrial to aquatic tetrapods [92] (Figure 4). Based on the Madeline robot design, researchers investigated how two to four flippers interact to modulate swimming performance in a self-propelled swimmer. Their results demonstrated that four flippers produce superior surge behaviour entailing high costs, whereas two flippers serve well during cruising at half the cost [92].

Ramesh et al. [93] proposed a biomimetic Mars robot inspired by extant and extinct animal features. In particular, the authors reported that the combination of bird-like feet, dinosaur-like legs, and a rat-like body led to a highly stabilised biomimetic robot. In 2010, German and co-workers [94] developed a robotic setup to simulate the burrowing behaviour for different bivalve shell shapes documented in the fossil record. The results lead to new knowledge of bivalve burrowing behaviour and the evolution of their functional morphology, as well as an inspired design for the construction of automatic burrowing devices [94]. 

In flight technology, Zakaria et al. [98] investigated the aerodynamic optimisation of the wing kinematics and planform of a designed pterosaur replica to minimise the cycle-averaged aerodynamic power and to maximise propulsive efficiency. The results led to interesting information on flapping pterosaurs in forward flight. Shahid et al. [99] reviewed the origin of avian flight considering the evolutionary, biophysical, and mechanical perspectives for its possible application in the designing of flying robots. The authors underlined how dynamic and kinematic models can support the study of extinct and extant organismal morphologies, leading to a better understanding of their structure and aerodynamic abilities, as well as a bio-inspired design of flying robots. The *Microraptor* was suggested as an interesting fossil model for biomimetic applications. Martin-Silverstone et al. [100] provided a comprehensive review on flight performance of fossil vertebrates, highlighting how recent discoveries can expand biomimetic designs to fossil taxa.

**Figure 5:**
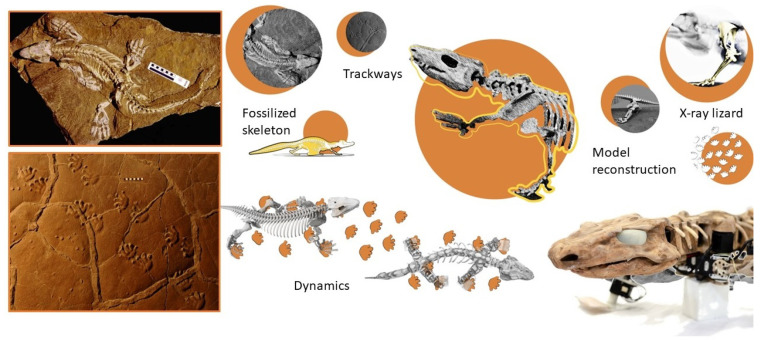
OroBOT, a moving robot model of the *Orobates pabsti* stem amniote based on CT scans of its skeletal remains and trackways. Image courtesy of Thomas Martens, Museum der Natur Gotha; Sebastian Voigt, Urweltmuseum Geoskop Thallichtenberg; Alessandro Crespi, EPFL Lausanne; John A. Nyakatura, Humboldt University, Berlin.

In a reverse biomimetics, fossil-based robotics can also help palaeontologists to unravel key knowledge of past life. In this regard, Nyakatura et al. [96] created “OroBOT”, a moving robot model of the *Orobates pabsti* stem amniote based on CT-scans of its skeletal remains (290-million-year-old fossils, Bromacker quarry, Thuringia, Germany, 2000) and preserved ancient footprints (Figure 5). In this way, they achieved quantitative data and interesting results on past amniote gaits. Song et al. [101] designed a bionic flapping wing mechanism to examine the aspects of potential aerodynamic performances in the proto wings of *Caudipteryx*. They quantitatively tested the lift and thrust, demonstrating that both are increased at higher flapping frequencies and thus verifying the ability of *Caudipteryx* to generate small aerodynamic forces. The same robotic model was previously used to investigate the aerodynamic effect of fixed wings during running [102].

Interesting examples were also provided by Long et al. [103] and Roberts et al. [104], using the robotic approach to test evolutionary hypotheses. 

Specifically, Long et al. [103] used physical simulations by means of autonomous robots to test the vertebrate adaptation hypothesis for which vertebrae evolution resulted in locomotor adaptation, stiffening body axis, and improved swimming performance. Successively, Roberts et al. [104] developed a population of early vertebrate jawless fish robot models to study vertebrae evolution. These robots were programmed as behaviourally autonomous, pursuing light as a proxy for feeding and avoiding hunting predators. Adaptive features, such as number of vertebrae, the span of the caudal fin, and the predator detection threshold (proxy for the lateral line sensory system) were planned to freely evolve. The results obtained supported the hypothesis that vertebrae also evolved as an adaptation for enhanced feeding and fleeing performances in early vertebrates.

## 4. Paleomimetics: Definition, Methods, and Tools

The review of the available literature provided in the previous section demonstrated how fossil-based inspirations have only recently been emerging. This is certainly due to recent technological advances in available instruments and numerical modelling that can now expand the biomimetic designs to fossil taxa. In this section, the definition, methods, and tools of Paleomimetics are presented with the intention to provide a conceptual framework for the development of a biomimetic design inspired by extinct species and evolution processes.

### 4.1. Definition

The “learning from nature” approach has been identified by numerous different terms, such as “biomimetics”, “bionics”, and “biomimicry” [1,2,3,4,5]. In 1957, the term “biomimetics” was coined by the engineer Otto Herbert Schmitt and was subsequently regulated and certified by the International Organization for Standardization (ISO 18458) in 2015 [105]. The term “bionics” was coined by the US Air Force Major J.E. Steele in 1960 based on a combination of the words “biology” and “technics”. The term “biomimicry” means imitation of the living and was coined by the natural scientist Janine M. Benyus in 1997 [1,2,3,4,5]. Over the past decades, other terms have been used in conjunction with this process, such as bio-inspiration, biomimesis, nature-based solutions, biologically inspired designs, and numerous others [1,2,3,4,5]. Each term slightly differs in objectives, principles, and approaches; however, they all describe a process based on the identification of a natural solution in the development of innovative technology.

Regarding the mimicry of extinct species, Shyam et al. [15] introduced the term Paleomimesis as “mimicking or learning from the past”. In her review, Tihelka [16] referred to “Palaeobiotechnology” as the potential industrial application of fossil-inspired technologies. In this article, the term “Paleomimetics” defines the imitation of extinct species and evolution processes based on the biomimetic [105] hybridisation with palaeontology. Specifically, the following definition is provided based on the adaptation of the biomimetic definition:

“Interdisciplinary cooperation between palaeontology and design approaches to solve practical problems through a functional analysis of extinct/extant biological systems and evolutive processes together with their abstraction and transfer into technological applications”. 

### 4.2. Methods and Tools

**Figure 6:**
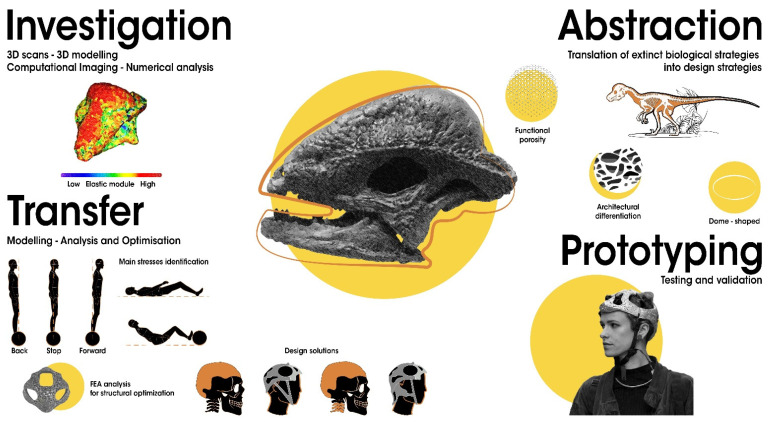
Paleomimetic process. A four-step bottom-up process of Paleomimetic solution transfer: from a fossil model to a technical application as exemplified by the *Pachycephalosaurus*-inspired helmet case study. (1) Extinct model identification and investigation: *Stegoceras validum* skull. (2) Abstraction of functional mechanisms in design principles: dome-shaped structure, architectural differentiation, and functional porosity determining lightness and resistance with energy-absorbing performances. (3) Transfer into models, analysis, and optimisation: 3D model of the helmet defining material, form, and structure; validation through numerical simulations identifying the principal stresses related to the final use; structural optimisation. (4) Prototyping, testing, and validation: prototype fabrication to be tested and validated. Design: Monica Bacelin. Research team: Carla Langella, Maddalena Mometti, Valentina Perricone, 2017. Graphical representation: Carmen Cerere. Image of the *Stegoceras validum* skull FEA analysis adapted from [111].

Variable biomimetic processes, models, and tools have been developed and described in the literature (see [5] for a review). The approaches can be divided into two main groups: *solution-based* or *problem-based* [4,5,20,67,106]. The first begins from the identification of adaptive functional solutions in biological species, followed by the recognition of the most suitable transfer in the design and technological areas. In the literature, this approach has also been defined as: *solution-based*, *solution-driven*, *biology push*, *biomimetics by induction* and *biology to design* [4,5,106]. The second approach begins from the analysis of a technical problem to the selection of biological models offering different novel solutions. This is also known as: *problem-driven*, *problem-based*, *challenge to biology*, *technology pull* and *biomimetics by analogy* [4,5,20,67,106]. In this article, a general bottom-up approach and supporting tools are simplified in four key steps; additionally, a preliminary case study on the *Pachycephalosaurus*-inspired helmet is used as an example (Figure 6). 

(1) *Extinct model identification and analysis*. The initial model is identifiable based on functional performance, supposed or already identified, or on an analogy of function and problem solving related to a defined final application: e.g., a helmet has the function of head protection, similar to the *Pachycephalosaurus* skull. The extinct model can be reconstructed, analysed, and interpreted using 3D scans, 3D modelling, computational imaging, and numerical simulations (see Section 2.2.1). Numerous studies regarding structures, functions, and performances of extinct species have already been carried out and can be taken into consideration for the development of new technical systems and processes. In the future, this step can be facilitated by the creation of a database concerning extinct species functionalities on par with the already existent biological databases, e.g., [5,106,107,108]. Regarding the *Pachycephalosaurus* case study, the functional capability of this skeletal structure has been biomechanically tested by palaeontologists using 3D reconstruction and FE analysis [109,110,111]. It explains the mechanical response of the robust, but lightweight, energy-absorbing trabecular structure in protecting the brain during the intraspecific head-butting behaviour, in which heads were used for fighting and defence [109,110,111]. 

(2) *Abstraction of functional mechanisms in design principles*. Once shapes and structures corresponding to adaptive solutions and functional principles have been recognised, it is possible to abstract and transfer them into the design of different technical products, according to the function similarities and innovation needs expressed by society. The abstraction process involves the translation of extinct biological adaptations into design strategies. The main aim of the abstraction is the description of how the extinct biological adaptation works without relying on the selected organism. Quick and simple drawings or diagrams illustrating features and mechanisms involved in the biological model can support the abstraction. They can also ensure an in-depth comprehension of the mechanisms lying behind the function and help fill gaps in palaeontological knowledge. Regarding the *Pachycephalosaurus* case study, the three identified design functional features were the dome-shaped structure, architectural differentiation, and functional porosity, which together determine lightness and resistance with energy-absorbing performances. Based on an analogy of function, the identified abstracted solutions suggested the development of a particularly lightweight helmet for speed accelerators in urban mobility (e.g., skateboards, hoverboards, and skates in general). Although not yet governed by precise regulations, these forms of transport necessitate head protection.

(3) *Transfer*. In this phase, the modelling, analysis, and optimisation of the artificial system based on the Paleomimetic design principle are developed. The technological implementation includes the choice of material, form, and structure supported by numerical simulations and validations, with particular attention and adaptation to their final use. The structural and functional extinct solutions are often neither the most advantageous nor the most adapted in any situation and context. Thus, a specific contextualisation and optimisation of the Paleomimetic technical solution is required. This step can be accomplished through an interdisciplinary collaboration using specific tools, such as “computer-aided optimisation”, knowledge databases, and algorithms [45,108,112]. In this regard, it is important to mention Claus Mattheck, who dedicated his research to an in-depth understanding of the mechanisms of biological growth adaptation and the modalities to simulate it in algorithms. He developed computer-aided optimisation (CAO) based on the growth of trees, bones, and other biological structures [113]. 

Regarding the *Pachycephalosaurus* case study, an inspired helmet was modelled integrating the identified solutions adapted to their specific final use. Helmets are composed by foam and shell: the first absorbs the most impact energies, whereas the second provides mechanical resistance and head protection, distributing impact loads on a wider foam area and increasing energy absorption capacity. The porous energy-absorbing trabecular system of the *Pachycephalosaurus* skull [111] was reproduced by using a very innovative and high-performance material, i.e., the foamed aluminium, a solid metal with gas-filled pores, which provides lightness and impact resistance comparable to a bone skeletal structure. Starting from this unique material, the *Pachycephalosaurus*-inspired helmet was designed with a dome-shaped structure leading to a very light, easily transportable, breathable, and aesthetically attractive head protector.

**Figure 7:**
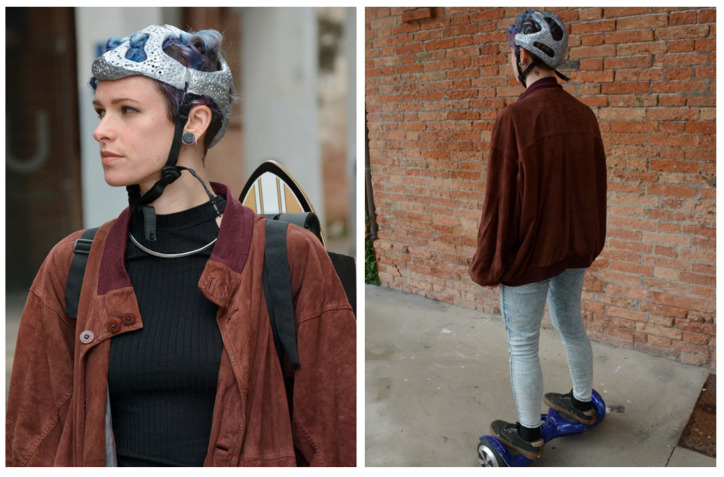
Pachycephalosaurus-inspired helmet. Design: Monica Bacelin. Research team: Carla Langella, Maddalena Mometti, Valentina Perricone, 2017.

(4) *Prototyping, testing, and validation* of the Paleomimetic artefact. The final step of the Paleomimetic process is the assessment of all calculated results by the fabrication of prototypes to be tested for final validation. In the reported case study, this step corresponds to the final realisation and validation of the *Pachycephalosaurus*-inspired helmet (Figure 7).

## 5. Paleomimetic Solutions: Promising Extinct Organismal Adaptations for Design Strategies

In this section, a series of promising functional Paleomimetic solutions is provided, based on available static and dynamic biomechanical studies (see Section 2.2.2). Different interesting structural solutions in fossil organisms emerge in palaeontological studies. Apart from the mechanical property of the material, organismal shape and structure can provide important solutions in the enhancement of mechanical strength. For example, different kinds of protective body armours have been developed in the animal kingdom, e.g., fish and pangolin scales, turtle carapaces, crocodiles, and armadillos’ body plates [114]. This armour consists of an external or superficial protection against biotic or abiotic environmental stresses in the form of overlapped, tasselled, interlocked, or fused rigid elements [69,114]. Always considered as a result of an evolutionary predator–prey arms race, body armour has been recently demonstrated to potentially fulfil other functional roles, such as thermal capacity [115]. The wide diversity in these structures and related high-performance mechanical behaviours, with respect to monolithic rigid structures, has attracted the attention of numerous researchers for the development of highly resistant biomimetic materials, structures, and devices, e.g., inspired by armadillo, nacre, fish scales, and diatoms [114,116]. Nonetheless, studies have demonstrated how extinct species body armour can provide interesting design solutions. As previously mentioned (Section 2.2.2), Du Plessis et al. [69,117] reported an interesting investigation on the armour of *Glyptodonts*. By means of micro-computed tomography, reverse-engineering, FE analysis, and mechanical testing of 3D printed models, the researchers demonstrated how the combination of high-density compact layers with a porous lattice core provide an optimised solution for increasing strength and high energy absorption capacities. As suggested by the authors, inspired by this adaptation, future protective structures could be produced using additive manufacturing. 

*Pachycephalosaurus* is a genus of pachycephalosaurid dinosaurs that lived during the Late Cretaceous Period in North America [110,111]. The genus name is derived from Greek and means “thick-headed lizard”, due to their distinctive head morphology with a dorsally solid bone thickened and dome-shaped cranium. As previously reported (see Section 4.2), the functional capability of this skeletal structure has been biomechanically tested by palaeontologists. Using 3D reconstruction and FE analysis [110,111], they explained the mechanical response of the robust but lightweight energy-absorbing trabecular structure in brain protection during the intraspecific head-butting behaviour [110,111]. Thus, the *Pachycephalosaurus* skull could inspire the development of new porous protective materials, structures, and devices.

Ammonoids are a group of extinct molluscs belonging to the same class of the extant genus *Nautilus* (Cephalopoda). The intersection of their septa with the external shell generates curves called suture lines. In ammonoids, these lines were highly complex and extensively frilled to be considerable as fractal curves. De Blasio [118] demonstrated that these complex suture lines were of aid in the counteraction of the external water pressure effect, diminishing shell shrinkage and improving buoyancy. As suggested by the author, this design could respond to “the challenging problem in the resistance of complex mechanical structures subjected to high pressure”.

Organisms experience a variety of movements (e.g., feeding, walking, swimming, and flying), adapting their structures to terrestrial, aquatic, and aerial environments [107,108]. Hence, during evolution, different anatomical adaptations, e.g., flexible muscles and complex architectural structures, were selected [119,120]. In particular, fascinating examples of feeding and flight mechanisms are here provided:

(i) In feeding mechanisms, placoderms are an interesting example. They are armoured fishes that lived during the Devonian Period, 415–360 million years ago. The largest of the placoderm was *Dunkleosteus terrelli*; it was a voracious predator equipped with powerful, bladed jaws characteristic of a novel ability to fragment prey prior to ingestion [121]. Anderson and Westneat [122] developed a biomechanical force and motion model of *D. terrelli* during feeding, showing a highly kinetic skull driven by a unique four-bar linkage mechanism. Taking into consideration a large sample of *D. terrelli* (about 6 m in length), they were able to estimate a maximal bite force of over 4400 N at the jaw tip and more than 5300 N at the rear dental plates. The authors declared that “this bite force capability is the greatest of all living or fossil fishes and is among the most powerful bites in animals”. This solution could inspire the development of novel jawed technical devices (e.g., robotic arms, biomedical devices, etc).
(ii) In flight mechanisms, the pterosaur provides interesting solutions and some of them have been already incorporated into flight technology (see Section 3). Indeed, Mesozoic pterosaurs were powered fliers with complex and multi-layered membranous wings supported by a single spar (the arm and an elongated fourth finger). Additional smaller membranes to the fore and the rear were also present for flight control [100]. The largest pterosaurs (10 m in wingspan, 250 kg, and 3 m head skulls) vastly exceeded any known flying animal in size and weight. Martin-Silverstone et al. [100] described in detail the key knowledge of flight in extinct and extant organisms suggesting a series of potential solutions for future flight technologies. They highlighted how “fossil forms can provide plethora structures and integrated systems that can contribute to next-generation aircrafts, robots, low-flutter fabrics, and ultra-light structures”. The authors suggested that pterosaur-based wing designs could lead to a novel method for achieving aeroelastic stability by optimising the control of aeroelastic flutter. They emphasised that their “actinofibril orientation, tissue layering, wing shape, and span-wise bone geometry” present in the wing had a crucial role in utilising and controlling aeroelasticity, which could be effectively transferred. New self-launching and landing robots can be inspired by the pterosaur’s ground launch ability; effective over a wide range of body size, this launch model can guide future improvements in unmanned aerial vehicle (UAV) launch, landing, and storage. Additionally, the authors suggested pterosaurs-inspired control surfaces and wings.

As already known, dynamic systems in nature are present even in sessile organisms, such as plants, which need to respond and use environmental biotic and abiotic features in the optimisation of their survival, growth, and reproduction [123,124]. Many plant movements are based on adaptive shape changes deriving from the hierarchical organisation of their materials and structures [123,124]. An example is pinecones that change their shape based on the hygroscopic expansion of their material (wood) and fibre orientation according to the degree of humidity. Indeed, the woody bract scales close in wet conditions (unfavourable for wind dispersal) and open under dry conditions to liberate their seeds. Interestingly, Poppinga et al. [125] reported this humidity-driven opening and closing motion in coalified conifer cones from the Eemian Interglacial and Middle Miocene. The author investigated the entire cone and separated seed scales of *Pinus* sp. and *Keteleeria* sp., showing distinct bending movements when transferred from a dry environment to a container filled with tap water. Micro-CT scans revealed the structural bilayer setup with sclereid layers, sclerenchymatous strands, and surrounding matrices, as seen in scales of extant pines. The authors stated that these biological, compliant mechanisms can be considered as “role models for resilient and maintenance-free biomimetic applications (e.g., adaptive and autonomously moving structures, including passive hydraulic actuators)”.

As an additional matter, the environmental contextualisation could be an important factor to take into consideration for a better identification of Paleomimetic solutions and fortiori considering that past species lived in totally different and even extreme climates than today. Indeed, as already underlined in Section 2.1.2, environmental context, origin, and historical evolution is a crucial aspect for understanding the form–function relationship in both extant and extinct organisms. In this regard, the field of paleoecology is specifically dedicated to the reconstruction of past environments in which organisms lived [126]. These studies consist in a reconstruction of abiotic (e.g., temperature, precipitation, water depth, and salinity) and biotic (e.g., types and numbers of species in a fossil assemblage) factors, beginning from a series of environmental proxies, such as: the analyses of organismal assemblage, tree rings, ice cores, paleoclimatic emulators, isotopic analyses of the sediment and its grain and texture [17,37]. These interpretations assume that past environments, processes, and organisms functioned similar to those of today [17,37]. Based on this set of assumptions and data, palaeontologists have developed different methods for inferring organismal function based on a direct analogy with living organisms (see EPB method Section 2.2.2). In this regard, Hickman [127] highlighted how also analogy with technical systems and processes can be used to further explore fossil form and to understand the functioning of past enigmatic organisms and structures. As an example, optic theory and analogy with lenses contributed to the acquisition of new insights regarding image formation and visual acuity of the trilobite eyes. This aspect could be particularly interesting for reverse biomimetics, and thus palaeontological studies could be further facilitated by future progress in Paleomimetics. 

## 6. Conclusions and Future Outlooks

In the course of time, biomimetic and bio-inspired design has attracted the attention of researchers from different scientific fields primarily taking into consideration extant species. Nonetheless, extinct species and evolutive processes reveal a great potential for biomimetic applications, thus motivating further investigation. This article represents the first comprehensive synthesis and starting point of a biomimetic design inspired by past organisms and processes, herein defined as Paleomimetics. Accordingly, an initial state of the art, definition, method, and tools are proposed all together with different key notions, conceptual framework, and promising functionally inspired strategies. These support the translation of morpho-functional fossil characteristics and processes into design solutions to meet emerging functional needs of contemporary lifestyles. In respect to the past biomimetic history, a Paleomimetic approach can presently emerge due to the advancement of palaeontological analytical techniques, e.g., image reconstruction, imagine analysis, 3D modelling and FEA. These technologies ensure high fidelity in the acquisition of extinct models, high reproducibility of complex geometry, and great reliability of results that can currently be reproduced using digital manufacturing techniques. Consequently, virtual palaeontology is now able to provide innovative solutions regarding the morphology, function, and evolution of fossils and extant species, leading to the new Paleomimetic approach. In a forthcoming future, the evolution of Paleomimetic design will require additional research for a systematic advancement of processes, methods, tools, and case studies. This could transform Paleomimetics into a new, affirmed biomimetic field. The realisation of a Paleomimetic database can be a useful support to implement this field, entailing a collection of extinct morpho-functional traits and emerging adaptations as well as biomimetic evolutionary framework, processes, and traps. Presently, this article represents a first attempt to set up a methodological approach and is an ongoing study for the authors. The efficiency of this approach will lie in the countless forms of hybridism between palaeontological notions and studies with biomimetic designs, leading to an innovative, adaptable, and programmable generation of evolute artefacts.
